# Supplementary material for: Novel Driver Strength Index highlights important cancer genes in TCGA PanCanAtlas patients
Source: PeerJ. 2022 Aug 11;10:e13860. doi: 10.7717/peerj.13860 (PMC9375969; doi:10.7717/peerj.13860)
Supplement: Supplemental Information 2 [file peerj-10-13860-s002.zip › COHORTS/genes plots/cohorts/2021_8_16_14_9_distribution_cohorts_males_DLBC.pdf]

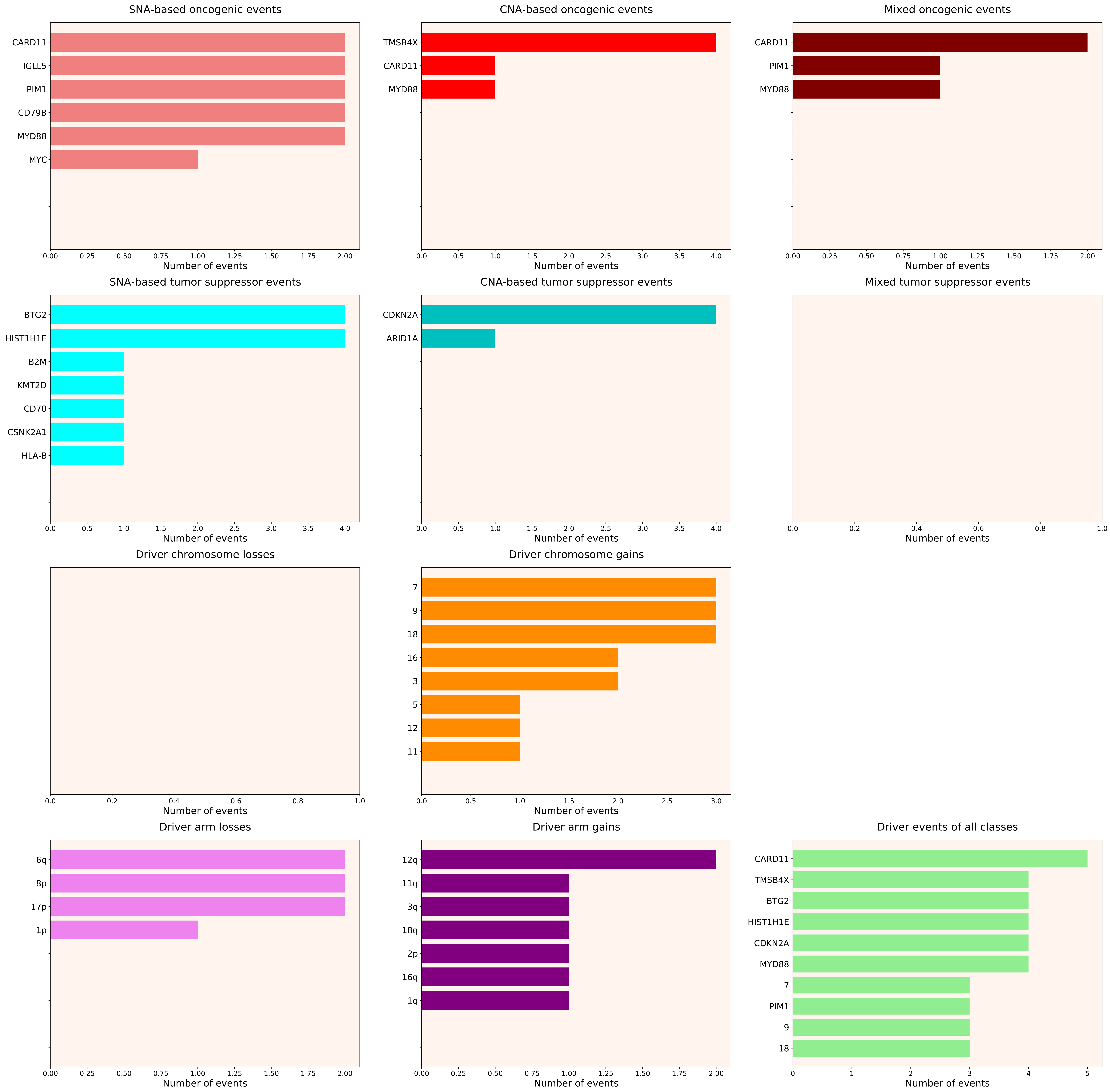
SNA-based oncogenic events
CARD11
IGLL5
PIM1
CD79B
MYD88
MYC
Number of events
CNA-based oncogenic events
TMSB4X
CARD11
MYD88
Number of events
Mixed oncogenic events
CARD11
PIM1
MYD88
Number of events
SNA-based tumor suppressor events
BTG2
HIST1H1E
B2M
KMT2D
CD70
CSNK2A1
HLA-B
Number of events
CNA-based tumor suppressor events
CDKN2A
ARID1A
Number of events
Mixed tumor suppressor events
Number of events
Driver chromosome losses
Number of events
Driver chromosome gains
7
9
18
16
3
5
12
11
Number of events
Driver arm losses
6q
8p
17p
1p
Number of events
Driver arm gains
12q
11q
3q
18q
2p
16q
1q
Number of events
Driver events of all classes
CARD11
TMSB4X
BTG2
HIST1H1E
CDKN2A
MYD88
7
PIM1
9
18
Number of events
